# Latency thresholds in telesurgery: are current clinical claims over-optimistic?”

**DOI:** 10.1097/MS9.0000000000004811

**Published:** 2026-03-03

**Authors:** Abdul Eizad Asif, Muhammad Hassaan Javaid, Muhammad Talha Sajid, Hasibullah Aminpoor

**Affiliations:** a Department of Medicine, Shalamar Medical and Dental College, Lahore, Pakistan; b Department of Medicine, Shifa College of Medicine, Islamabad, Pakistan; c Faculty of Medicine, Kabul University of Medical Sciences “Abu Ali Ibn Sina”, Kabul, Afghanistan

*To the Editor*,

Telesurgery, also known as robotic surgery conductor through high-speed telecommunication networks, is amongst the most evolving areas of modern surgical technology frontiers. Since the successful transcontinental telesurgery that led to the validation of the usability of telesurgery over thousands of miles, technology in robotic surgery has developed significantly with advancements in imaging capabilities, motion scaling, and Artificial Intelligence-based decision support^[^1^]^. Further development in telesurgery programs with standardized training courses in robotic surgery has also assisted in training experts across the globe in this technology^[^2,3^]^. Recent developments in 5G communication technology and fiber optic communication have decreased the time gap and made telesurgery ready to change the technological face of modern surgery in order to make specialty surgical facilities available to all, leaving no geographical hurdles in their way, especially in rural areas^[^4–6^]^.

Nevertheless, despite these technological advancements, “latency measured as the time between a surgeon’s control command and the corresponding action performed by the robot remains the single most important technical issue that presently affects telesurgery safety and accuracy.” Latency was found to directly influence dexterity, synchronization, and mental task performance. Some important studies have established that there is a substantial loss of accuracy once a threshold of delay is crossed^[^7^]^. Later studies carried out on simulators have established specific delay levels above which a tolerance threshold is acceptable. Delays of sub-200 ms values protect task performance, but at a delay of about 300 ms, this is mostly feasible. However, above a delay of either 500 ms or 700 ms, there is a substantial drop-off in precision^[^8,9^]^, and further studies using animal subjects continue to validate these ranges, which are also confirmed by successful task accomplishment within a delay range of about 220–320 ms, past which there is adverse effect on suturing and manipulation of tissues^[^10^]^. However, studies continue to corroborate that telesurgery could accomplish a task with an equal measure of precision as conventional surgery, despite varying amounts of delay^[^6^]^.

In considering the confirmation of these assertions with the evidence that actually exists, the importance of the preconditions of scalability in the real world, infrastructure variety, and surgeon accommodation has been noted as an area still waiting for further investigation through systematic reviews^[^6^]^. Although contemporary robots are able to integrate the effects of small lag times with predictive motion analysis and adaptive control loops, these are still in the process of being developed and have had little opportunity for validation^[^5^]^. There is also great interest in the universal implementation of telesurgery that must be tempered with the realities of availability and the effects of variation on networks. Times under 200 ms are ideal for preserving the natural synchronization of the hands and the eyes, 200–320 times may be manageable with optimal implementations, but beyond that range, the clinical validity starts becoming important. Therefore, in light of the increasing focus on digital surgery reported recently in Annals of Medicine and Surgery, the discussion surrounding latency thresholds calls for a re-evaluation. It thus seems that although significant progress has been achieved with telesurgery technology, the proportionate interface between the claim and the evidence documented is imbalanced.

We confirm that no AI tools were utilized in the conduct or writing of this manuscript, in accordance with the TITAN checklist^[^11^]^.

## Data Availability

Not applicable.
